# Rape

**Published:** 1867-10

**Authors:** R. P. Hunt

**Affiliations:** Chicago


### RAPE.—WHAT IS IT?

By R. P. Hunt, M.D., Chicago.

Frequently, in reading the different journals, from all parts of the country, we find reported cases of rape. Now, Mr. Editor, I wish to ask some of your many readers:—Is rape possible? A strong man may violate a child, but can any man, of no matter what strength, violate a woman? Does not a woman who is raped, as said, more or less yield? Is not the passion which would cause an animal in the human shape, so much excited as to attempt such an outrage, is he not, I ask, so excited that, before the crime could be committed, nature would involuntarily relieve itself?

Does not a brave and defiant woman always defend nerself, and also that which is dearest to her, her virtue? Is there nothing in the old Joe Miller joke, in which a rape was committed by a small man upon a large woman, both standing:—"Oh! your Honor, I stooped." Are there not many stooping rapes? Did not Queen Elizabeth exemplify this case well, when she was appealed to? "Hand me your sword," said she to the officer. The sword was handed. "No, I only wish the scabbard; keep the sword." The sovereign's orders were obeyed. Queen Betty with the scabbard, the officer with .the blade, she orders him to place the sword in the scabbard. At each effort to do so, the great Ruler of England would twist her wrist and move the mouth of the scabbard. "Why do you not replace the blade?" says Queen Bess. "Your Majesty moves the scabbard too much," was the reply. "Well, if the woman had done the same, she might probably have been more fortunate." Except by brute force, used by more than one, can rape be accomplished. Is not fright or willingness a necessary accompaniment? Is not rape like seduction, where one must meet the other.

